# Reduced body weight is a common effect of gene knockout in mice

**DOI:** 10.1186/1471-2156-9-4

**Published:** 2008-01-08

**Authors:** Danielle R Reed, Maureen P Lawler, Michael G Tordoff

**Affiliations:** 1 Monell Chemical Senses Center, Philadelphia, USA

## Abstract

**Background:**

During a search for obesity candidate genes in a small region of the mouse genome, we noticed that many genes when knocked out influence body weight. To determine whether this was a general feature of gene knockout or a chance occurrence, we surveyed the Jackson Laboratory Mouse Genome Database for knockout mouse strains and their phenotypes. Body weights were not available for all strains so we also obtained body weight information by contacting a random sample of investigators responsible for a knockout strain.

**Results:**

We classified each knockout mouse strain as (1) lighter and smaller, (2) larger and heavier, or (3) the same weight, relative to control mice. We excluded knockout strains that died early in life, even though this type of lethality is often associated with a small embryo or reduced body size. Based on a dataset of 1,977 knockout strains, we found that that 31% of viable knockout mouse strains weighed less and an additional 3% weighed more than did controls.

**Conclusion:**

Body weight is potentially a latent variable in about a third of experiments that use knockout mice and should be considered in interpreting experimental outcomes, e.g., in studies of hypertension, drug and hormone metabolism, organ development, cell proliferation and apoptosis, digestion, heart rate, or atherosclerosis. If we assume that the knockout genes we surveyed are representative then upward of 6,000 genes are predicted to influence the size of a mouse. Body weight is highly heritable, and numerous quantitative trait loci have been mapped in mice, but "multigenic" is an insufficient term for the thousands of loci that could contribute to this complex trait.

## Background

The mechanisms underlying the control of body weight are undoubtedly complex but there have been few attempts to assess exactly how complex. In order to gauge the number of pieces in the body weight puzzle, we attempted to estimate the number of genes involved. One way to do this is to study the effects on body weight of knocking out all mouse genes, one by one. Something similar has been done in yeast and worms but for the mouse this is probably years and perhaps decades away [1,2]. Therefore, we focused on the information that was available: the Mouse Genome Database (MGD), a database of knockout strains and their phenotypes created and maintained by the Jackson Laboratory [3,4]. In mice, about 10% of known genes have been nullified, the effect of the alleles studied, and the results deposited in this on-line compendium. We conducted a survey of the MGD, with the goal to estimate the proportion of genes in the mouse that contribute to body size.

## Results

**Figure 1:**
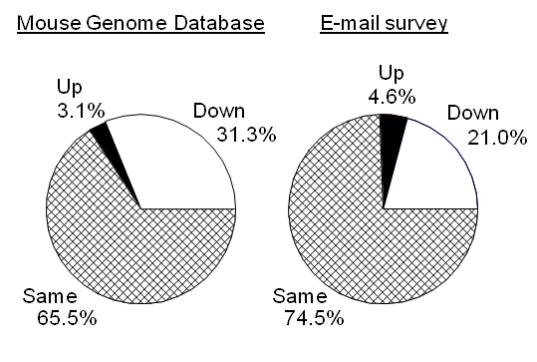
The proportion of viable mouse knockout strains that have one of three body weight outcomes relative to a comparison group: increased, decreased, or unchanged. The chart on the left illustrates data extracted from an on-line database (Mouse Genome Database) that describes the characteristics of mouse knockout strains, and the chart on the right summarizes a survey by e-mail of investigators who initially did not describe body weight of knockout strains but provided the information when queried.

The data set contained information about 1,977 knockout strains. Of the viable knockout strains with body weight information, 65.5% were reported to have no difference in body weight, 31.3% had reduced body weight, and 3.1% had increased body weight compared with a reference group ([EQ2 in Methods]; Table 1, Figure 1). These differences were not due to the genetically mixed background or to congenic islands because knockout mice on a uniform genetic background had slightly higher rates of body weight phenotypes than did mice on a mixed background (38% versus 32%).

**Table 1:** Categorization of gene knockout effects on body weight in mouse strains

Category	KO strains (N)
Nonviable	542
No BW info	1078
BW same	234
BW reduced	112
BW increased	11
N total strains	1,977

KO = knockout; BW = body weight. Same = body weight the same as an appropriate control group, e.g., littermates. For a description of strains selected for study and the definition of "nonviable," see the text.

About half of the entries had information about body weight, and 27% of the entries involved strains that were not viable [EQ3 in Methods]. Although "nonviable" knockout strains often had small embryos or neonates, these are not included in the totals for reduced body weight. A few genes were knocked out more than once but whether we included or excluded these multiple knockout strains made little difference to the outcome. Of the 105 follow-up inquiries we made by e-mail, 43 investigators responded with information that allowed us to categorize the body weight of the knockout strain, 39 investigators did not respond, 12 e-mail addresses were undeliverable, and 11 investigators responded but were unable to provide specific information about mouse body weight, or the knockout strain was determined to be nonviable by our definition. Of the 43 usable responses, nine knockout strains had reduced body weight (21.0%), two had increased body weight (4.6%), and the remainder did not differ in body weight (74.4%). These frequencies did not differ from those obtained in the survey of the MGD (X^2^_(2) _= 2.1, NS). There was no tendency for knockout strains studied in the year 2000 or later to be different than those studied earlier, either in body weight or in viability (p > 0.05).

Assuming that the information contained in the MGD for knockout strains for the six chromosomes we surveyed is representative of the 25,613 genes in the total mouse genome [5], we estimate that 6,916 genes are indispensable [25,613 genes × 0.27, proportion of genes that are indispensable], 5,852 genes decrease body weight when nullified [18,697 dispensable genes × 0.313, proportion of dispensable genes that reduce body weight], and 580 genes increase body weight [18,697 dispensable genes × 0.031, the proportion of dispensable genes that increase body weight].

## Discussion

The observation that null alleles of mouse genes often reduce but sometimes increase body weight is not new [6]. However, to our knowledge, no previous work has examined the general effect of gene knockout on mouse body weight because these types of knockout studies are undertaken to address specific research questions about particular genes. To impartially evaluate the general effect of gene knockouts on body weight, we read every Mouse Genome Database record for every knockout gene on six chromosomes and noted the remarks made about body weight. Based on this analysis, we estimate that more than 6,000 genes could contribute to mouse body size. Of the genes with null alleles that are dispensable, ~30% result in a mouse with reduced body weight and another 3% result in a mouse with increased body weight relative to mice with an intact gene. The genome is biased toward weight gain, with 10 times more genes increasing body size than decreasing it. This observation is consistent with the suggestion that mice are "hard-wired" to favour positive energy balance [7].

This survey was an observational study, and the caveats of this approach are important to consider. One limitation was the lack of information for almost half the strains surveyed; however, we tentatively suggest that the lack of information does not appreciably bias the results. The pattern of results was similar whether the data were present in the MGD or obtained through a follow-up poll. Another point to consider is that genes are not selected for knockout randomly, but are made as a "cottage industry" by investigators for their own purposes [8]. Therefore, we emphasize that the 10% of the genes listed in the MGD are unlikely to be a representative sample of all genes. However, we assessed changes over time and found that the frequency of phenotype (body weight or lethality) did not change. There are no obvious trends for the first knockout strains to have more body weight phenotypes than the later ones. We excluded from the reduced body weight category all knockout strains that did not survive past weaning. Many of these lethal strains are small during their development, and had we included them in the estimates of genes that reduce body weight, the number of "body weight genes" would be much higher. This decision is conservative in the sense that it is reasonable to suppose that while null alleles of a particular gene might be lethal, hypomorphs of the same gene might be viable but with a reduced body weight. These points together suggest that the results reported here are more likely to be under- than overestimates of the number of body weight genes.

Our estimate of the number of genes that are indispensable to normal mouse development is larger than others. For instance, the Knockout Mouse Project [9], an international effort to establish a repository and database for knockout mouse strains, estimates that 15% of genes are indispensable, compared with the 27% we identified in this survey. Our estimate is also almost twice as high as comparable values for flies, worms, and yeast [10-13]. Mice may be more genetically fragile than flies, worms, or yeast and therefore null mutations in the mouse may be more consequential; or, the selection of genes for knockout in the mouse may bias the results toward the most indispensable genes, as mentioned above. It could also be that requiring that the mice survive until weaning increases this percentage of indispensable genes beyond that of worms, flies, and yeast, which do not have a comparable developmental period.

Another issue to consider in this study is the diet that mice were fed and how this may have exacerbated or masked body weight effects. Investigators studying body weight and obesity will often try feeding an energy-dense diet if the null allele has no effect when mice are first tested on a standard chow diet. If knockout mice do not gain weight when fed this diet, they are deemed to be "resistant to dietary obesity." It would be worthwhile to know how many of the knockout strains not specifically made to study obesity would differ in body weight if fed a high-fat diet. In yeast, systematic removal of each gene and testing with a variety of nutrients and feeding conditions indicates that up to 40% of yeast strains with a missing gene have a growth phenotype [14]. Likewise, a larger percentage of mouse knockout strains might have altered body weight compared to control mice if fed high-calorie diets.

The results of this survey indicate the number of null genes that *can *affect mouse body weight, and not the number of naturally occurring alleles that actually *do *affect body weight. One might wonder whether it is reasonable to call a gene with a man-made null allele a "body weight" gene if there is no naturally occurring allele of comparable severity in ordinary mice. The utility of this definition of "body weight gene" depends in part upon the particular scientific question addressed. For network biologists, this approach of gene-by-gene knockout is not only valid, but desirable. The existence of a null (or severe hypomorphic) allele is less important than whether the gene participates in some way in the development or maintenance of body size. However, geneticists may find this particular concept of "body weight gene" troublesome: if a gene is not allelic in a mouse population then it may be of little or no interest to those who study the inheritance of body weight. These two views can be reconciled by understanding that any gene that can be nullified in the laboratory can potentially have a comparable allele in the mouse population, and thus the issue is reduced to one of allele frequency; i.e., how often do null alleles arise and persist? We cannot currently estimate the number of genes that are naturally allelic and that contribute to body size in mouse populations, but this survey does set the upper limit for the number of single genes with null alleles that can contribute to it.

The implications of these data extend beyond the question of gene networks and individual differences in body size. A sizable proportion of knockout mice generated to understand particular traits suffer from the side effects of altered body weight and its consequences. Without appropriate controls, these results may confound simple interpretation; the results attributed to a specific effect of the gene might instead be due to general effects related to lower body weight. Investigators have become keenly aware of this type of problem and have developed several methods to remedy it, e.g., conditional and/or tissue-specific knockout. Whether more control over knockout gene expression lessens the general effect on body weight is not known. Regardless of the advances in knockout technology, any naturally occurring null alleles in mice will function like the knockout strains studied here: the gene will be null in all tissues in which it is expressed, and null at every developmental stage.

We made no attempt in the course of this survey to quantify the degree of body weight change of knockout mouse strains relative to control groups of mice and this is a noteworthy limitation of this type of survey methodology. For some knockout strains, the small size of the mice is obvious, and often investigators did not quantify the reduction, reporting that the mice were "small" or "stunted" relative to controls. In some cases, the weight differences are reported as a percentage of wild-type, e.g., "10% smaller than control mice," but without statistical tests. About one-tenth of the genes that affect body weight when nullified resulted in a larger rather than a smaller mouse, and this result provides a rough estimate of the number of negative feedback genes. A precise measure will require that all knockout strains of mice be evaluated with standardized data collection procedures.

One more caveat is that not all of the effects of gene knockout necessarily are due to the nullified gene itself. The embryonic stem cell line used to make the construct is often from one of the 129 mouse strains, and the resulting knockout strain is often a chimera of C57BL/6J (B6) and 129 DNA [15]. Some investigators have proposed exploiting this feature of knockout strains to map QTLs [16], but this situation creates a problem for the interpretation of mouse knockout studies. If the null allele is on a mixed genetic background, it is not clear whether any observed trait differences between the knockout and wild-type groups are due to the null gene itself or to the background genotype. Even after extensive backcrossing, an influential 'congenic footprint' of flanking DNA can remain [17]. This point is especially relevant to the study of body weight because there are many QTLs among the common strains used to construct knockout mice [18-21]. However we found that knockout strains on a genetically uniform background had a slightly *higher *rate of body weight change compared with those on a genetically mixed background. Thus it is unlikely that these types of effects can account for the majority of weight change in knockout mice observed in this survey.

Our interest in this survey began when we were evaluating genes as candidates under a linkage peak and noted many more knockout genes with a body weight phenotype than we expected. An issue in complex genetics is the extent to which significant linkage peaks are composed of one gene with an allele of large effect, or many genes with smaller effects that are so close together that they cannot be distinguished by statistical means. These survey results do not resolve this puzzle but they do suggest that the original observation that knockout strains often have reduced body weight was accurate. This realization has led us to reassess from less to more likely the possibility that multiple genes contribute to one QTL peak. Furthermore, if about one-third of viable genes when knocked out reduce or increase body weight, knockout experiments alone may not provide convincing evidence to validate candidate genes for body weight suggested by QTL analysis. Understanding how body weight is determined by this network of genes presents an extraordinary challenge. A first step is defining the scale of the question, and this survey has answered this question, albeit in a preliminary way.

## Conclusion

Thirty-one percent of viable knockout mouse strains weighed less and 3% weighed more than control mice, indicating that changes in body weight among knockout mouse strains are common. Extrapolating from these results, many more genes contribute to mouse body weight than suggested by other approaches.

## Methods

### Extracting and coding of database records

The data used in this study were obtained through query of the MGD and by an e-mail survey of investigators who deposited information about knockout mice but did not mention body weight in their original report. Entries were extracted from the MGD *Phenotype and Alleles *sub-database on August 15, 2006. We confined the search to all records (as defined below) on a random subset of chromosomes (1, 2, 7, 16, 18, and 19). All published phenotype information for knockout mouse strains was originally extracted by workers at the Jackson Laboratory and catalogued in the database, and the summary of this information is referred to as a record. For each chromosome in our selected subset, we read each record for every knockout strain, and the information provided about body weight was coded by one of us (MPL). Strains were eliminated from consideration if they were double knockouts, if the null allele disrupted adjacent genes, or if the strain was heterozygous, i.e., with one intact and one null allele.

Because this database captures information provided by individual investigators, the degree and type of information about body weight and body size varied across records. Each knockout strain on the final list (N = 1,977) was placed into one of five categories: (a) nonviable, (b) no change in body weight, (c) reduced body weight, (d) increased body weight, or (e) no information about body weight. Because this categorization relies on decisions about diverse types of information, here we provide a detailed description of the categorization decisions so that this analysis can be repeated by other investigators.

Category (a) included knockout strains that did not survive past weaning (nonviable). Strains described as "embryonic lethal" were included in this category, as well as mice that were born alive but died shortly after birth. We also included in this category strains in which mice were born alive but fewer than 60% survived to weaning with routine care. The word "most" was interpreted to mean more than 60%, so a strain that was described by the phrase "most mice died before weaning" was categorized as nonviable. Sometimes investigators prolong the life of the mice by hand-feeding them or use other types of support, and we also classified those strains as nonviable.

The remaining viable mouse strains for which there was body weight information were classified into category (b), (c), or (d). If investigators specifically remarked that the body weight was the same or if they reported that mice were normal or indistinguishable from the relevant control mice, the strain was classified as category (b). In other words, if an investigator reported that "knockout mice were normal," we included the strain in category (b). If the mice were smaller at any point in their development, the strain was included in category (c). This is an important point because one pattern occasionally mentioned by investigators was for the knockout mice to be small early in life but to catch up or nearly catch up as they aged. Category (d) included strains of knockout mice that were larger on average than mice from a reference group.

To reduce errors, a second investigator (DRR) categorized a subset of strains, and these decisions were compared and were found to be consistent. We also noted (a) the year in which the knockout phenotype report was published to assess the pattern of lethality and body weight effects over time and (b) whether the knockout strain was on a uniform or genetically mixed background.

### Surveying missing information that may bias estimates of gene knockout effects

Investigators create knockout mice to address specific research questions often unrelated to body size, so sometimes no mention is made of the body weight. To understand how these missing data might affect the conclusions of this survey, we contacted a subset of these investigators to request details about body weight. We chose 105 entries at random that were reported by the investigator as viable mice but that did not have body weight information. We obtained the depositor's e-mail address either from MGD records or by searching the Internet. An e-mail message was sent that briefly explained the survey, with a request for information about the knockout strain's body weight. The depositor's responses were read by one of us (MPL) and coded following the strategy described above. We compared the proportion of knockout strains reported to be the same, reduced, or increased in body weight from the original MGD records with the data obtained by e-mail survey using a Χ^2 ^test, with p < 0.05 as the criterion for significance.

### Estimating the effect of gene knockout on body weight

First, we computed the number (N) of viable strains with body weight information [EQ1]. Using this value, the proportions of viable strains with unchanged (b), decreased (c), and increased (d) body weight were computed [EQ2]. The proportion of category (a) (nonviable) knockout strains was calculated [EQ3] and was used as an estimate of the proportion of indispensable genes:

*Category b + c + d = N viable knockout strains with body weight information*

*[(Category c) ÷ (N viable knockout strains with body weight information)] *

*= proportion of knockout strains with reduced body weight*

*[(Category a) ÷ (Category a + b + c + d + e)] = proportion of nonviable knockout strains*

We used these proportions to extrapolate to the mouse genome and estimate the total number of genes for which null alleles can change body weight. These calculations were done in two steps using the total number of known genes estimated by the most recent mouse genome annotation [5]. In EQ4A, the proportion of indispensable genes was calculated by multiplying the total number of known genes by the proportion of nonviable knockout strains (taken from [EQ3]). The number of dispensable genes was obtained by subtracting the number of indispensable genes from the total number of genes [EQ4B]. In EQ5, the number of dispensable genes (taken from [EQ4B]) was multiplied by the proportion of null alleles that reduce body weight (taken from [EQ2]). Similar calculations were computed for increased and unchanged body weight.

*[Total number of genes × proportion of nonviable knockout strains] = N indispensable genes*

*[Total number of genes - N indispensable genes] = N of dispensable genes*

*[N dispensable genes × proportion of knockout strains with null allele that reduced body weight] = N genes that reduce body weight when the allele is null*

One concern is that the strains listed in the database are not representative and thus biased because the most interesting genes have been knocked out first. To determine whether there was a change over time, we used a Χ^2 ^test to compare the proportion of mice in each category for reports published before the year 2000 with those published in 2000 or later (these two groups had roughly the same number of records).

## Abbreviations

MGD Mouse Genome Database.

## Authors' contributions

MGT conceived and designed the experiments. MLP performed the experiments. MLP and DRR analyzed the data. MGT and DRR wrote the paper.
